# ggsashimi: Sashimi plot revised for browser- and annotation-independent splicing visualization

**DOI:** 10.1371/journal.pcbi.1006360

**Published:** 2018-08-17

**Authors:** Diego Garrido-Martín, Emilio Palumbo, Roderic Guigó, Alessandra Breschi

**Affiliations:** 1 Centre for Genomic Regulation (CRG), The Barcelona Institute for Science and Technology, Barcelona, Spain; 2 Universitat Pompeu Fabra (UPF), Barcelona, Spain; 3 Institut Hospital del Mar d’Investigacions Mèdiques (IMIM), Barcelona, Spain; Johns Hopkins University, UNITED STATES

## Abstract

We present ggsashimi, a command-line tool for the visualization of splicing events across multiple samples. Given a specified genomic region, ggsashimi creates sashimi plots for individual RNA-seq experiments as well as aggregated plots for groups of experiments, a feature unique to this software. Compared to the existing versions of programs generating sashimi plots, it uses popular bioinformatics file formats, it is annotation-independent, and allows the visualization of splicing events even for large genomic regions by scaling down the genomic segments between splice sites. ggsashimi is freely available at https://github.com/guigolab/ggsashimi. It is implemented in python, and internally generates R code for plotting.

## Author summary

Efficient visualization of splicing events from RNA sequencing data is key for the computational analysis of alternative splicing. This process, that results in a single gene giving rise to multiple transcripts, is usually illustrated through sashimi plots: a representation of the read coverage and the support of each splicing junction in the region of interest. However, available tools for this purpose present several limitations that significantly hinder their applicability. Among them, dependence on event annotation, visualization difficulties with moderate sample sizes and long introns, use of non-standard file formats or inefficient implementations. With ggsashimi, we comprehensively overcome these flaws and provide the user with a fast, stand-alone application that generates publication-ready sashimi plots. Furthermore, ggsashimi supports the display of aggregated experiments, a crucial feature in order to explore alternative splicing in the era of large RNA sequencing projects.

This is a *PLoS Computational Biology* Software paper.

## Introduction

Alternative splicing is the process through which different combinations of exons of the same gene are selected to produce a variety of mature coding and non-coding transcripts [1]. The genome-wide landscape of alternative splicing can be easily profiled by RNA sequencing (RNA-seq) and tens of thousands of different RNA-seq experiments are now publicly available. While visualization of RNA-seq data is crucial for exploratory data analysis, visualization of splicing events is currently not dynamically integrated in common genome browsers, and stand-alone software are annotation-dependent.

Visualizing splicing events is particularly challenging because such events usually occur between two regions, known as splice sites, that are not contiguous on the genome sequence, and can be as distant as tens or even hundreds of kilobases in linear space. The representation of a splicing event implies drawing a connective element that illustrates the presence of a splice junction between two splice sites. The sashimi plot [2] is a very effective and established diagram which combines the information of read coverage along a gene –a signal track– with curves connecting splice sites supported by RNA-seq data.

A tool for drawing sashimi plots was initially developed as part of the MISO suite [3], a software that quantifies and compares alternative splicing from RNA-seq experiments. Current popular implementations include a stand-alone utility to create sashimi plots specifically for MISO-indexed splicing events [2] and a built-in available within the Integrative Genomics Viewer, IGV [4]. Thus, the former relies on a proper compatible annotation of the event, while the latter requires IGV installation and the time-consuming uploading of voluminous alignment files. Moreover, both of them represent splicing events for each RNA-seq experiment on a separate line, which hinders the comparison of more than a dozen samples.

## Design and implementation

Like the original tool for sashimi plots [3], the data processing part of ggsashimi is implemented in python. In contrast to the original tool, ggsashimi internally generates an R script which uses the ggplot2 library [5] for the graphical rendering. To ensure reproducibility, it is distributed in a Docker image, which includes the ggsashimi python script and all the required dependencies.

In its simplest usage, ggsashimi generates a publication-ready image with a read coverage histogram and curves connecting splice sites, from a single RNA-seq experiment. Curves have variable widths, proportional to the relative number of reads supporting the splice junction. In line with the most utilized bioinformatics file formats, the required input is a standard alignment BAM file (with no special aligner-dependent features), and genomic coordinates indicating the region to display. The BAM file must be coordinate-sorted and indexed in order to efficiently extract the reads from a determined genomic region. Splice junctions are inferred directly from the BAM file, and no prior knowledge of the splicing event is needed. The output of ggsashimi is available in both vector (SVG, PDF) and raster (PNG, JPEG, TIFF) formats. For the latter, the resolution in pixels per inch can be defined by the user.

To allow comparisons across multiple experiments, a list of files can be specified and the signal for each experiment is plotted on a separate line. However, with increasing number of samples, visual comparison of separate plots becomes too overwhelming and some form of aggregation is essential. To this end, ggsashimi can aggregate data for hundreds of experiments and represent plots only for the aggregated measures (see Features).

Finally, an annotation plot is optionally generated to visualize transcript structures in the specified region. Again, in line with current standards, a Gene Transfer Format (GTF) file is required, with no additional description of the splicing events. Because splicing events often involve short exons flanked by proportionally very large introns, the genomic regions included between two splice sites (inferred from the alignments and not from the annotation) can be optionally shrunk for better graphical representation. We observed that updating the length of the splice junctions to the original length raised to the power of 0.7 usually renders a good balance between the lengths of introns and exons.

### Features

ggsashimi presents several unique features that distinguish it from its predecessors and make it a useful tool especially for large-scale projects:

1. Annotation-independent: no need for annotation of the splicing events.
2. Stand-alone command-line tool: no need for cpu-expensive applications (e.g. IGV).
3. Scales for a large number of samples by multiple aggregation methods:
  - overlay: the signal of each individual sample is placed upon the others, using transparency to enhance visualization. The number of reads supporting each event are shown for all samples. Transparency can be modified by tweaking the parameter --alpha. This is suitable when the number of samples per group is relatively small (≤10).
  - mean: the mean signal and the mean number of reads supporting each event across individual samples are shown.
  - median: the median signal and the median number of reads supporting each event across individual samples are shown. Both mean and median number of reads supporting an event can also be displayed in combination with the signal overlay.
4. Focuses on informative regions, by compressing the length of long intronic segments with no splicing events.

## Results

**Fig 1:**
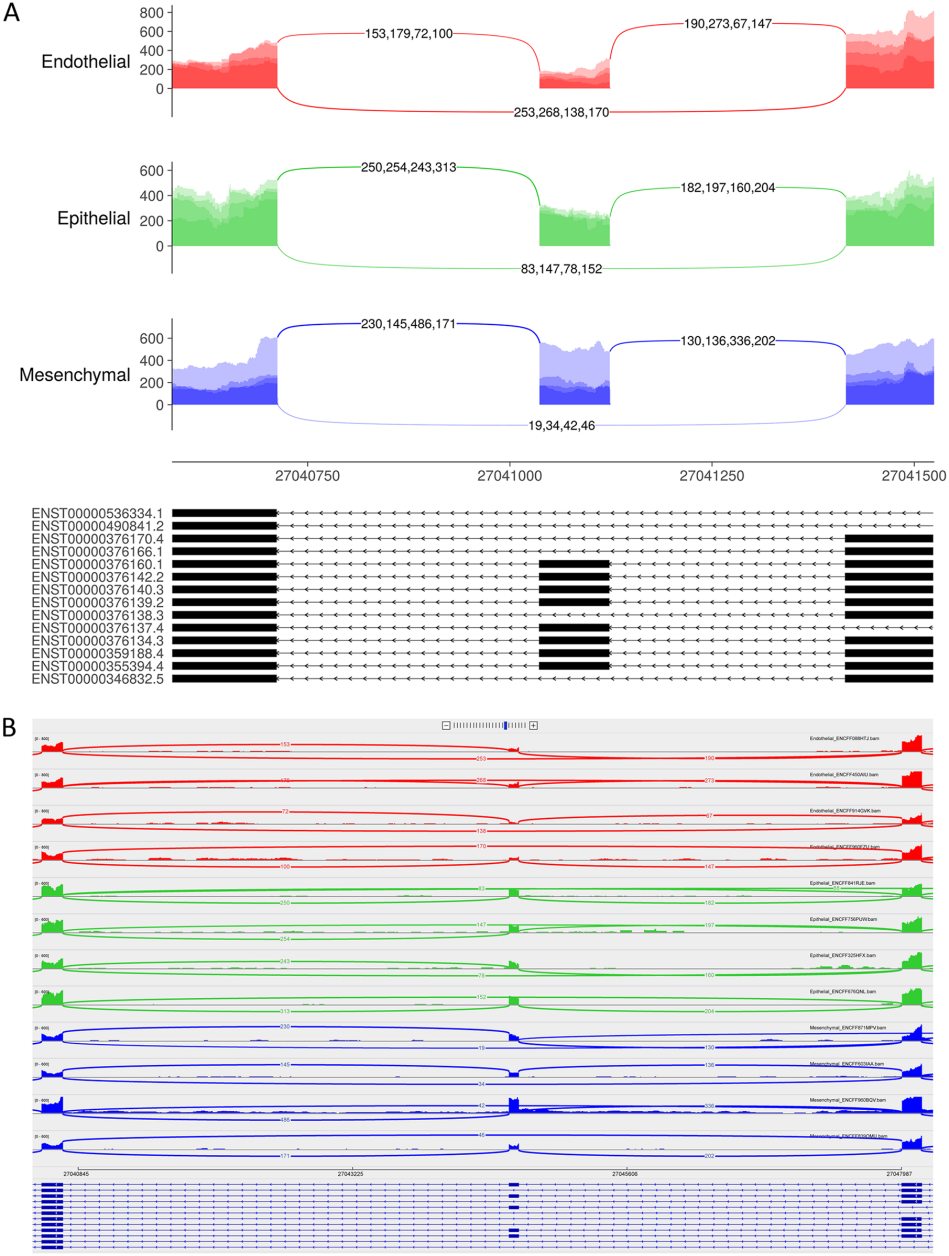
Comparison of sashimi plots generated by ggsashimi and IGV. Sashimi plots of 12 ENCODE samples belonging to 3 cell type groups (endothelial, epithelial and mesenchymal) for the region chr10:27040584-27048100 obtained by ggsashimi (A) and the sashimi-plot utility within IGV (B). The inclusion level of the exon chr10:27044584-27044670 is clearly higher in mesenchymal cells (blue), followed by epithelial (green) and endothelial cells (red). While this trend is barely observable in the IGV sashimi, which becomes complex and confusing with multiple samples, as it makes one sashimi plot per sample; the --overlay option of ggsashimi allows aggregating samples belonging to the same groups, providing a much better overview of the event. In addition, the presence of long introns flanking the exon of interest substantially enlarges the connective elements and hinders visualization in IGV sashimi. Conversely, ggsashimi avoids this problem thanks to its --shrink option, which updates the original intron lengths, enhancing visualization.

To illustrate how ggsashimi performs and to compare it with existing implementations, we obtained a set of 12 RNA-seq samples from the ENCODE project [6], publicly available at www.encodeproject.org. Samples were classified into three cell type groups: endothelial, epithelial and mesenchymal. We focused on a single cassette exon (chr10:27044584-27044670) with different levels of inclusion across the three cell type groups (mesenchymal > epithelial > endothelial). For comparison purposes, the genomic region containing the selected splicing event was represented both using ggsashimi and the sashimi-plot built-in available within the IGV Browser (Fig 1). In the case of ggsashimi, aggregation of samples belonging to the same group (through the --overlay option) and shrinkage of intron lengths were applied (see Features), enhancing the visualization of the event.

## Availability and future directions

Although the sashimi representation for splicing events is one of the standards for splicing visualization, current implementations present several limitations that narrow substantially its application. We believe that our implementation solves many of the current issues, especially we eliminated the need for specialized annotation formats and we support summarized views for hundreds of samples. Since ggsashimi uses the most popular file formats and has very few dependencies, it can be easily integrated in any splicing analysis pipeline, and can facilitate the interrogation of alternative splicing in large-scale RNA sequencing projects, such as ENCODE [6] and GTEx [7]. ggsashimi is freely available at https://github.com/guigolab/ggsashimi. Further extensions of ggsashimi include incorporating spread metrics to accompany mean and median aggregating methods, allowing the user to select which type of reads to plot (e.g. uniquely mapped) or optionally showing only the aggregated coverage.
